# Self-efficacy and oral health outcomes in a regional Australian Aboriginal population

**DOI:** 10.1186/s12903-022-02471-0

**Published:** 2022-10-17

**Authors:** Eleanor Jane Parker, Dandara Gabriela Haag, Andrew John Spencer, Kaye Roberts-Thomson, Lisa Marie Jamieson

**Affiliations:** University of Adelaide, Adelaide, Australia

**Keywords:** Self-efficacy, Oral health, Aboriginal, Indigenous

## Abstract

**Background:**

Perceived self-efficacy has been associated with psychological well-being, health behaviours and health outcomes. Little is known about the influence of self-efficacy on oral health outcomes for Aboriginal adults in Australia, a population experiencing high levels of oral health conditions. This study examines associations between oral health-related self-efficacy and oral health outcomes in a regional Aboriginal Australian population and investigates whether the associations persist after adjusting for sociodemographic characteristics and other general and oral health-related psychosocial factors.

**Methods:**

Cross-sectional data were obtained from the baseline questionnaire of the Indigenous Oral Heath Literacy Project, South Australia. Oral health-related self-efficacy was measured using a six item scale, with total sum scores dichotomised into high/low self-efficacy. Oral health outcomes included self-rated oral health and oral health impacts, measured using the Oral Health Impact Profile (OHIP-14). Generalized linear models with a log-Poisson link function were used to estimate Prevalence Ratios (PR) of poor self-rated oral health according to levels of oral health-related self-efficacy. Multivariable linear regressions were used to estimate the association between oral health-related self-efficacy and OHIP-14 scores. Blocks of confounders were subsequently added into the models, with the final model including all factors.

**Results:**

Complete data were available for 252 participants (63%) aged 18 to 82 years (mean age of 37.6 years). Oral health-related self-efficacy was associated with poor self-rated oral health, with a 43% (PR = 1.43 (95% CI 1.09, 1.88)) greater prevalence of poor self-rated oral health among those with low self-efficacy. Oral health-related self-efficacy was associated with OHIP-14 severity scores, with a score over six points higher for those with low self-efficacy (B = 6.27 95% CI 2.71, 9.83). Although addition of perceived stress into the models attenuated the relationship, associations remained in the final models.

**Conclusion:**

Lower levels of oral health-related self-efficacy were associated with a higher prevalence of poor self-rated oral health and greater impacts of oral health among Aboriginal adults in regional South Australia. These associations persisted after controlling for sociodemographic and psychosocial confounders, suggesting that increasing self-efficacy may provide an opportunity for improving oral health outcomes for Aboriginal adults.

## Background

Oral health is fundamental to overall health and wellbeing. Oral conditions affect quality of life, with physical, social and psychological impacts in addition to economic consequences for individuals and communities [1]. In Australia, disparities in oral health exist, with Aboriginal and Torres Strait Islander Australians suffering a greater burden of oral disease and impacts of oral health than non-Aboriginal Australians [2, 3]. Aboriginal and Torres Strait Islander Australians, hereafter referred to as Aboriginal to identify with the traditional owners of the lands on which this study was conducted, make up 3% of the Australian population [4]. Improving oral health outcomes for Aboriginal adults is essential to improving overall health and wellbeing. Achieving this requires a more in-depth understanding of the issues impacting on the oral health of Aboriginal adults to enable more specific and culturally safe interventions to be developed. One area warranting further investigation is the relationship of psychosocial factors and oral health outcomes. Psychosocial factors are considered a crucial factor contributing to poor health and oral health and may be critical in understanding the oral health needs of more vulnerable populations [5].

A key psychosocial dimension related to health and oral health outcomes reported extensively in the literature is perceived self-efficacy, with an individual’s perceived self-efficacy shown to influence a broad range of health-related behaviours [6]. Self-efficacy is a core element of Bandura’s Social Cognitive Theory [7], with Bandura defining self-efficacy as the “conviction that one can successfully execute the behaviour required to produce the outcome” [8]. Self-efficacy is also a key feature of the Health Belief Model [9], with self-efficacy acting directly and indirectly on health behaviours and therefore health outcomes. In terms of general health, self-efficacy has been associated with psychological well-being and predicts self-care and health-related quality of life for people with chronic health conditions, included cardiovascular disease, diabetes, multiple sclerosis and arthritis [10–14].

Perceived self-efficacy assesses an individual’s belief in their ability to have control over their own behaviours and therefore their ability to engage in healthy behaviours irrespective of other external and internal factors. For example, a common approach to evaluating an individual’s perceived self-efficacy involves asking how confident a person is that they will perform a certain behaviour when they are stressed, busy or tired [15, 16]. This suggests that the association between self-efficacy and oral health outcomes may still persist after other psychosocial factors are accounted for. Furthermore, a number of studies have suggested that factors such as perceived stress play an important role in shaping the general and oral health at a population level in Australia [17–19]. To the best of our knowledge there is only one study that investigated the association between self-efficacy and oral health outcomes for Aboriginal Australians, accounting for general psychosocial confounders. The study included a sample of women pregnant with an Aboriginal baby, and showed that self-efficacy persisted as a risk indicator for poor self-rated oral health after adjusting for a range of general and oral health-specific psychosocial factors [20].

We therefore aimed to address this gap in the literature pertaining to self-efficacy and oral health outcomes for Aboriginal adults specifically in regional South Australia. This Aboriginal community resides in a regional centre of around 13, 800 residents, with just over 18% (2500 adults and children) identifying as Aboriginal or Torres Strait Islander [21]. This Aboriginal community experiences social and economic disadvantage, with higher rates of unemployment and lower income than other areas of Australia [22]. Higher rates of impact of oral conditions [23] and higher rates of poor self-rated oral and general health than the Australian population [24] are also documented. Understanding the relationship between self-efficacy and oral health outcomes can be used to develop a deeper understanding of the precursors of oral health among Aboriginal Australians, enabling improved design and targeting of preventative interventions. We sought to examine associations between oral health-related self-efficacy and oral health outcomes in a regional Aboriginal Australian population and investigate whether the associations persist after adjusting for sociodemographic characteristics and other general and oral-health specific psychosocial factors.

Specifically this study aimed to:

- describe the prevalence of high/low oral health-related self-efficacy according to sociodemographic characteristics;
- investigate whether oral health-related self-efficacy is associated with oral health impacts and self-rated oral health;
- if the association in (b) was supported, determine if associations between oral health related self-efficacy and oral health outcomes persisted after controlling for sociodemographic and other psychosocial characteristics of perceived stress, perceived coping and oral health-related fatalism.

We hypothesised that study participants with low self-efficacy would report more oral health impacts and poorer self-rated oral health, and that these associations would remain after controlling for sociodemographic and other psychosocial characteristics.

## Methods

Data were obtained from the Indigenous Oral Health Literacy Project (IOHLP), a randomised controlled trial utilising a delayed intervention design [25] based in South Australia. This paper pertains to cross-sectional analysis of baseline data, collected in October and November 2010, for a convenience sample of 400 Aboriginal adults. Based on previous research with this community, recruitment methods included self-nomination, referral, word of mouth and visits at local community centres [26]. Eligibility criteria consisted of being Aboriginal or Torres Strait Islander, over 18 years of age, and living in Port Augusta or nearby communities. Questionnaires were completed as an interview, self-complete or a combination of both, as determined by the participant. Recruitment and administration of questionnaires was managed by project officers with local community cultural knowledge. Utilisation of these recruitment methods, and the approach to administration of the questionnaire, were deemed essential elements to ensure cultural acceptability. The project officers were provided with a scripted method of introducing and administering the questionnaire.

The exposure of interest, oral health-related self-efficacy (OH-SE), was measured using six items adapted from a self-efficacy scale developed by Finlayson and colleagues [16]. The six items asked participants to rate how confident they felt about their ability to brush their teeth at night when they were: (a) under a lot of stress; (b) depressed; (c) anxious; (d) feeling like they did not have the time; (e) tired and (f) worried about other things in their life. Responses were on a Likert scale scored with 1 = not at all confident, 2 = hardly ever confident, 3 = occasionally confident, 4 = fairly confident, and 5 = very confident. Based on feedback from expert and Aboriginal advisory groups, an additional response option of “I never feel like this” was added, and treated as a missing response. Internal consistency was high (Cronbach’s alpha = 0.93). Responses were summed to give a possible scale score of 6–30, so that higher scores indicate higher OH-SE. The psychometric properties of the OH-SE scale have been assessed and reported for this community [27]. Scores were dichotomised into high (above the median score of 20) and low (at the median or lower) OH-SE.

Oral health outcomes included (1) the self-reported impact of oral health conditions using the shortened form of the Oral Health Impact Profile (OHIP-14) [28], previously validated among Aboriginal populations [29], and (2) self-rated oral health (SROH). For OHIP-14, responses to each item were summed to create an OHIP-14 severity score, with high scores representing higher reported impacts. For SROH, participants were asked to rate their dental (or oral) health with response options of “excellent”, “very good”, “good”, “fair” and “poor”. Responses were dichotomised to “excellent, very good and good” and “fair and poor”, with those as “fair and poor” classified as having poor SROH.

**Fig. 1:**
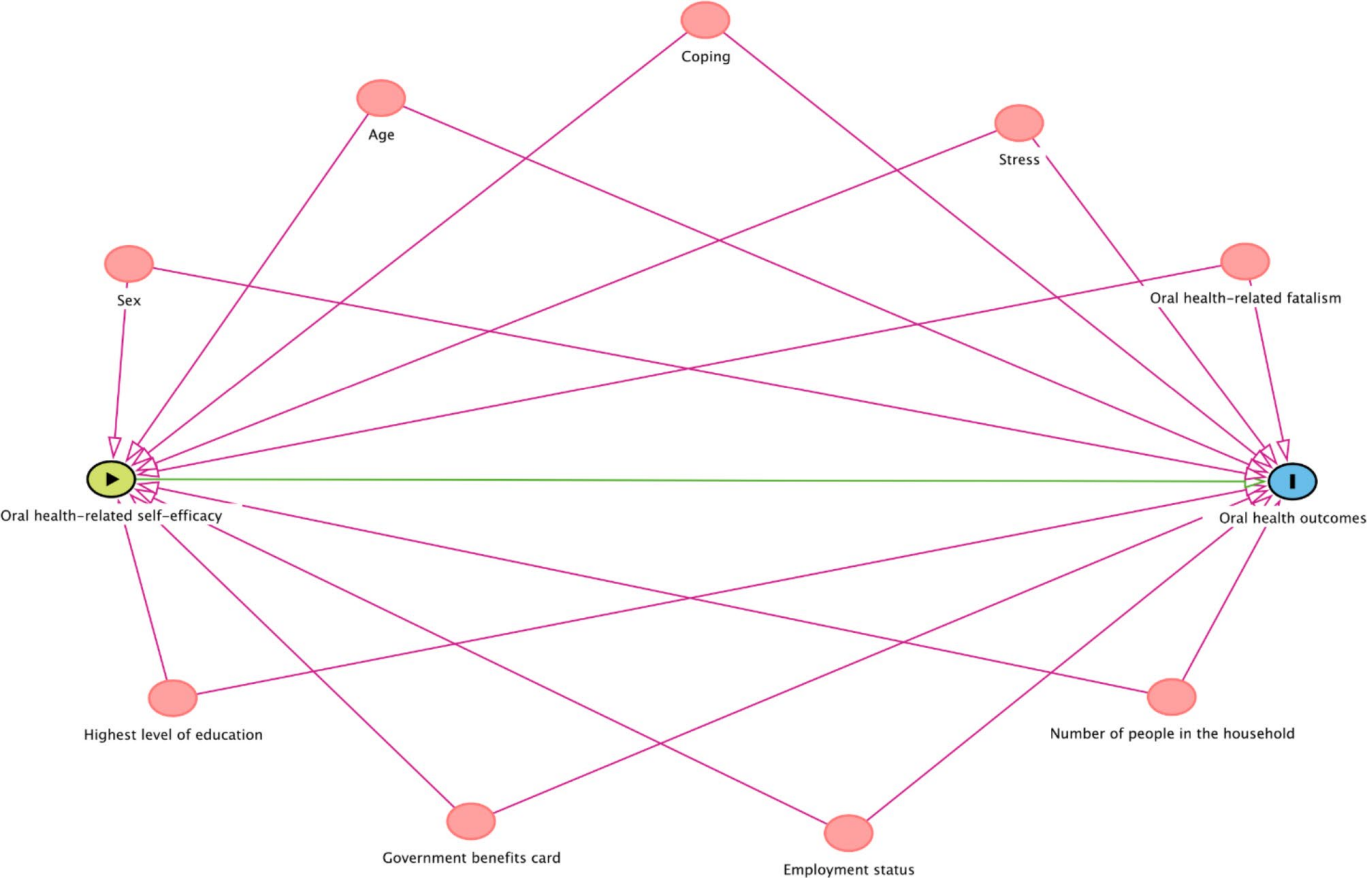
Direct Acyclic Graph (DAG) for the association between oral health-related self-efficacy and oral health outcomes

Confounding factors were identified based on the literature and theoretical associations with both exposures (OH-SE) and oral health outcomes [30–32], as depictured on the Direct Acyclic Graph (DAG) Fig. 1. These confounders were grouped according to (a) demographic characteristics (age and sex), (b) socioeconomic factors (level of education attained (dichotomised to include “no schooling, primary or high school” and “trade, TAFE or University”), employment status (“unemployed or other” and “paid employment”), ownership of a Government Concession Card (“yes” or “no”), and number of people staying in the house on the previous night (dichotomised to “5 or more” and “4 or less”)), and (c) psychosocial factors of perceived stress and coping, and oral health-related fatalism (OH-F).

Perceived stress and coping were measured using an adapted Perceived Stress Scale (PSS), developed by Cohen and colleagues [33] and used extensively in the international literature [17, 34, 35]. The PSS aims to measure the degree to which individuals perceive life situations as stressful, with 7 items reflecting distress and 7 items reflecting coping. For the purposes of this study, each item asked participants to reflect on how they have felt during the last year, for example, “how often in the last year have you felt either nervous or stressed?” reflecting distress, and “how often in the last year have you felt you were on top of things” reflecting coping. Responses to all items were on a five-point scale ranging from (0) not at all, to (4) very often. The psychometric properties of the PSS have been assessed among Aboriginal and/or Torres Strait Islander people [36]. Their study indicated that an adapted version (a-PSS13) was culturally appropriate for Aboriginal and/or Torres Strait Islander populations after the exclusion of one item (“How often during the LAST YEAR have you dealt well with life hassles). For this current study we therefore utilised the adapted Perceived Stress Scale (a-PSS13). Responses for each sub-scale were summed, with possible scores ranging from 0–28 for perceived stress, so that high scores reflected higher levels of distress, and possible scores for perceived coping ranging from 0–24 with higher scores reflecting higher levels of coping.

Oral health-related fatalism (OH-F) was based on a single item used by Finlayson and colleagues [16]. In this current study, five items were generated based on the range of health conditions prevalent in this community, and asked participants to indicate their level of agreement: “most people will… (1) eventually develop problems with their teeth; (2) need to have their teeth pulled out; (3) eventually get a toothache; (4) have bleeding gums; and (5) get wobbly teeth”. Response options were on a Likert scale scored from 1 (strongly disagree) to 5 (strongly agree). Based on feedback from expert and Aboriginal advisory groups an additional response option of “I don’t know” was added, treated as a missing response. Internal consistency was high (Cronbach’s alpha 0.88). Responses were summed to give a scale score ranging from 5 to 25, with high scores reflecting high oral health-related fatalism. The psychometric properties of the OH-F scale have reported for this community [27].

### Analytic methods

All analyses were conducted for a complete case sample. Descriptive analyses were performed, including distribution of participants according to the exposure, confounding factors and outcomes.

Generalized linear models with a log-Poisson link function and robust standard errors were used to estimate Prevalence Ratios (PR) and their 95% confidence intervals (CI) of poor SROH according to levels of OH-SE. Adjusted PRs and their respective 95% CIs were assessed after blocks of confounders were added into the models. The final model included all factors.

Multivariable linear regressions were used to estimate the association between OH-SE and OHIP-14 scores, using Beta coefficients and their 95% confidence intervals (CI). Blocks of confounders were subsequently added into the models, with the final model including all factors.

Sensitivity analysis was conducted to identify if including those with the median score in the high or low OH-SE group impacted on the results. The sensitivity analysis confirmed that patterns of associations remained for both approaches. Analyses were carried out using STATA 15.0.

## Results

Complete data were available for 252 participants (63%) aged 18 to 82 years and a mean age of 37.6 years (95% CI 35.7, 39.4). Table 1 shows the sociodemographic characteristics of the sample. More than two thirds were female, one quarter had a level of education including a trade, TAFE or university, less than one quarter were in paid employment and just over 85% owned a government benefits card. The mean OH-SE score (range 6–30) was 20.2 (95% CI 19.3, 21.1). The mean OH-F score (range 9–25, median 23) was 21. 7 (95% CI 21.3, 22.1). Perceived stress scores ranged from 0 to 28, with a mean of 14.2 (95% CI 13.5, 14.9). Perceived Coping ranged from 0 to 24 with a mean of 11.9 (95% CI11.3, 12.4). The mean OHIP-14 severity score was 21.4 (95% CI 19.6, 23.2), and almost half the participants (47.2% 95% CI 31.1, 53.4) rated their oral health as *fair or poor* (poor SROH).

**Table 1:** Distribution of sample characteristics, confounders and outcomes

	Percent (95% CI) or Mean (95% CI)
**Age group**	
18–24	23.8 (18.9, 29.5)
25–34	24.6 (19.7, 30.3)
35–49	29.8 (24.4, 35.7)
50–82	21.8 (17.1, 27.4)
**Sex**	
male	30.6 (25.2, 36.5)
female	69.4 (63.5, 74.8)
**Highest level of education**	
Trade, TAFE or university	24.2 (19.3, 29.9)
none, primary or high school	75.8 (70.1, 80.7)
**Employment status**	
paid employment	23.4 (18.6, 29.1)
unemployed/other	76.6 (70.9, 81.4)
**Government benefits card**	
no	14.3 (10.5, 19.2)
yes	85.7 (80.8, 89.5)
**Number of people in the house on previous night**	
4 or less	55.6 (49.3, 61.6)
5 or more	44.4 (38.4, 50.7)
**Perceived stress** (Mean, 95% CI)	14.2 (13.5, 14.9
**Perceived coping** (Mean, 95% CI)	11.9 (11.3, 12.4)
**Oral health fatalism** (Mean, 95% CI)	21. 7 (21.3, 22.1).
**OHIP-14 severity** (Mean, 95% CI)	21.4 (19.6, 23.2)
**Poor self-rated oral health** (prevalence, 95% CI)	47.2 (31.1, 53.4)

Table 2 shows the distribution of high and low efficacy according to sociodemographic characteristics. The proportion of participants with low OH-SE did not vary by sex. Among those in the oldest age group, 40% had low self-efficacy, compared with just over 57% in the 35–49 year age group. Among those in paid employment there were nearly 20% less participants with low OH-SE. One third of participants without a benefits card had low OH-SE. Table 2 also shows the mean scores for perceived stress, perceived coping, and OH-F for those with high and low OH-SE. For those with low OH-SE, the mean stress score was just over three units higher than those with high efficacy. Mean scores for perceived coping and OH-F did not vary across high and low efficacy groups. The mean OHIP-14 severity score was six units higher among those with low efficacy than for those with high efficacy.

**Table 2:** Oral health-related self-efficacy according to sociodemographic characteristics and psychosocial confounders

	Oral health self-efficacy Row % (95% CI) or Mean (95% CI)	
	**High OH-SE**	**Low OH-SE**
**Sex**		
male	48.1 (37.1, 59.2)	51.9 (40.8, 57.7)
female	49.7 (42.3, 57.1)	50.3 (42.9, 57.7)
**Age**		
18–24	51.7 (39.1, 64.0)	48.3 (36.0, 60.9)
25–34	45.2 (33.3, 57.7)	54.8 (42.3, 66.7)
35–49	42.7 (32.0, 54.1)	57.3 (45.9, 68.0)
50–82	60.0 (46.6, 72.1)	40.0 (27.9, 53.4)
**Highest level of education**		
trade, TAFE or university	42.6 (30.8, 55.3)	57.4 (44.7, 69.2)
none, primary or high school	51.3 (44.2, 58.4)	48.7 (41.6, 55.8)
**Employment status**		
paid employment	59.3 (46.4, 71.2)	40.7 (28.9, 53.6)
unemployed/other	46.1 (39.2, 53.2)	53.9 (46.8, 60.8)
**Government benefits card**		
no	66.7 (49.9, 80.1)	33.3 (19.9, 50.1)
yes	46.3 (39.7, 53.0)	53.7 (47.0, 60.3)
**Number of people in the house**		
4 or less	55.0 (46.7, 63.1)	45.0 (36.9, 53.3)
5 or more	42.0 (33.2, 51.3)	58.0 (48.7, 66.8)
**Perceived stress** (Mean, 95%CI)	12.6 (11.7, 13.6)	15.7 (14.7, 16.6)
**Perceived coping** (Mean, 95%CI)	12.0 (11.2, 12.9)	11.7 (11.0, 12.5)
**Oral health-related fatalism** (Mean, 95%CI)	21.8 (21.2, 22.4)	21.6 (21.0, 22.2)

Table 3 shows the distribution of oral health outcomes according to sociodemographic characteristics and by levels of OH-SE. The mean OHIP-14 severity score varied by age and sex, with females having a score nearly five units higher than males, and participants in the second highest age group having a score 12 units higher than those in the youngest age group. Those without paid employment reported more oral health impacts, with a score over five units higher than those in paid employment. The prevalence of poor SROH was lowest among the youngest participants, with 18.5% and 30.4% less participants rating their oral health poorly than in the 25–34 year age group and 35–49 year age groups respectively. Those with low self-efficacy had higher OHIP-14 severity scores. Among those with low efficacy, 55.5% had poor SROH, around 17% relatively more than for those with high efficacy.

**Table 3:** Distribution of oral health outcomes according to sociodemographic characteristics and levels of self-efficacy

	OHIP-14 severity Mean (95% CI)	Poor SROH Row % (95% CI)
**Sex**		
male	18.1 (14.8, 21.3)	42.9 (32.3, 54.1)
female	22.9 (20.7, 25.0)	49.1 (41.8, 56.5)
**Age**		
18–24	14.6 (11.5, 17.7)	28.3 (18.4, 41.0)
25–34	22.1 (18.9, 25.2)	46.8 (34.7, 59.2)
35–49	27.1 (23.4, 30.9)	58.7 (47.2, 69.3)
50–82	20.3 (16.6, 24.0)	52.7 (39.6, 65.5)
**Highest level of education**		
trade, TAFE or university	20.5 (16.9, 24.1)	52.5 (40.0, 64.6)
none, primary or high school	21.7 (19.6, 23.8)	45.5 (38.6, 52.7)
**Employment status**		
paid employment	17.2 (13.5, 21.0)	52.5 (39.9, 64.9)
unemployed/other	22.7 (20.6, 24.7)	45.6 (38.7, 52.7)
**Government benefits card**		
no	18.8 (13.2, 24.3)	47.2 (31.7, 63.3)
yes	21.9 (19.9, 23.8)	47.2 (40.6, 53.9)
**Number of people in the house the previous night**		
4 or less	21.4 (18.9, 23.9)	47.1 (39.0, 55.5)
5 or more	21.4 (18.7, 24.1)	47.3 (38.2, 56.6)
**Oral health-related self-efficacy**		
high	18.2 (15.7, 20.8)	38.7 (30.5, 47.6)
low	24.5 (22.1, 27.0)	55.5 (46.7, 63.9)

Oral health-related self-efficacy was associated with poor SROH, with over 40% (PR = 1.43 (95% CI 1.09, 1.88) greater prevalence of poor SROH among those with low OH-SE (Table 4). When sociodemographic characteristics were added into the model, low OH-SE was associated with 1.49 higher prevalence of poor SROH (PR = 1.49 (95% CI 1.14, 1.96) than for those with high OH-SE. When perceived stress was added into the model, the prevalence of poor SROH was 1.40 times higher among those with low OH-SE than among those with high OH-SE (PR = 1.40 (95% CI 1.06, 1.86). Adding perceived coping and OH-F (models 4 and 5 respectively) had little reduction on the prevalence ratio for poor SROH.

**Table 4:** Unadjusted and adjusted prevalence ratios for poor self-rated oral health

	Prevalence ratios for poor self-rated oral health (95% CI)					
	Unadjusted	Model 1	Model 2	Model 3	Model 4	Model 5
**Self-efficacy**						
High	Ref	Ref	Ref	Ref	Ref	Ref
Low	1.43 (1.09, 1.88)*	1.47 (1.13, 1.92)*	1.49 (1.14, 1.96)*	1.40 (1.06, 1.86)*	1.39 (1.05, 1.84)*	1.38 (1.04, 1.84)*

*p < 0.05

Model 1: age and sex

Model 2: Model 1 + socioeconomic factors (level of education, employment status, government concession card and number of people in the household)

Model 3: Model 2 + Perceived Distress

Model 4: Model 3 + Perceived Coping

Model 5: Model 4 + Oral health-related fatalism

Oral health-related self-efficacy was associated with OHIP-14 severity scores, with a score over 6 units higher for those with low OH-SE (B = 6.27 95% CI 2.71, 9.83) (Table 5). Addition of demographic characteristics into model 1 and socioeconomic factors into model 2 had little impact on the association between low OH-SE and oral health impacts (model 2: B = 6.22 95% CI 2.68, 9.77). When perceived stress was added in model 3, the Beta coefficient reduced from 6.22 to 4.03, an absolute attenuation in the OHIP-14 score of 2.24 units (B = 4.03 95% CI 0.52, 7.53). This corresponds to a 35% decrease in the strength of association between low OH-SE and oral health impacts. Addition of perceived coping and OH-F resulted in no real further reduction.

**Table 5:** Unadjusted and adjusted associations for oral health-related self-efficacy with OHIP-14 severity

	Beta coefficient (95% CI)					
	Unadjusted	Model 1	Model 2	Model 3	Model 4	Model 5
**Self-efficacy**						
High	Ref	Ref	Ref	Ref	Ref	Ref
Low	6.27 (2.71, 9.83)*	6.56 (3.09, 10.03)*	6.22 (2.68, 9.77)*	4.03 (0.52, 7.53)*	3.73 (0.20, 7.25)*	3.96 (0.45, 7.47)*

*p < 0.05

Model 1: age and sex

Model 2: Model 1 + socioeconomic factors (level of education, employment status, government concession card and number of people in the household)

Model 3: Model 2 + Perceived Distress

Model 4: Model 3 + Perceived Coping

Model 5: Model 4 + Oral health-related fatalism

## Discussion

This study assessed associations between OH-SE and subjective measures of oral health among a regional Aboriginal population in South Australia. Levels of OH-SE varied by age and some, but not all, socioeconomic variables. The prevalence of poor SROH was greater among those with lower OH-SE. Adjusting for confounders attenuated the relationship. Perceived stress had the most notable impact on the relationship between OH-SE and OHIP-14 scores, however, in the final model higher levels of oral health impacts remained for those with lower efficacy beliefs.

The association between levels of self-efficacy and oral health outcomes is an important finding adding to the developing body of literature demonstrating the importance of psychosocial determinants of oral health for Aboriginal Australian populations. This finding is consistent with that among pregnant Aboriginal women in South Australia, whereby low self-efficacy persisted as a risk indicator for poor self-rated oral health after controlling for a range of sociodemographic and psychosocial confounders [20]. The OH-SE items used in both studies asked only about a participant’s confidence that they would brush their teeth at night when feeling a range of emotions and in various psychological states, and not about any other health behaviours or health beliefs. Despite the focus on tooth brushing, the association with oral health outcomes is important to further develop our understanding of the role of efficacy beliefs in oral health, specifically for the Aboriginal population. Higher levels of self-efficacy can increase the likelihood of oral health promoting behaviours [37, 38], with some evidence that self-efficacy can be improved with focussed interventions and support for chronic disease self-management, as well as preventive health behaviours [39–42]. Interventions to improve self-efficacy may improve oral health outcomes for populations at high risk of poor oral health.

For both outcome measures, the addition of perceived stress into multivariable models resulted in the most substantial attenuation in the association with OH-SE. While this was modest for the prevalence of poor SROH, the reduction in the association with the OHIP-14 severity score was 2.24 units, a relative attenuation of 35%. This indicates that perceived stress is an important psychosocial factor to consider when investigating determinants of oral health for Aboriginal people. Despite this role of perceived stress, OH-SE remained significant in all models, indicating that even among more highly stressed individuals, self-efficacy is likely to be an important factor in evaluating oral health outcomes. This is consistent with the findings for pregnant Aboriginal women in South Australia, with a group of psychosocial factors including perceived stress, attenuating the odds of poor SROH by 17% [20]. This is an area that warrants further study to determine the impact that oral health specific self-efficacy has on the relationship between perceived stress, a general psychosocial measure, and oral health outcomes. If oral health-related self-efficacy has a protective effect in modifying the relationship between stress and oral health outcomes, interventions that improve an individual’s perceived self-efficacy may conceivably have the most impact for those who experience higher levels of stress.

The weaknesses of this study must be acknowledged and interpretation of findings assessed in light of the small sample size and, in particular, the high proportion of study participants excluded from this analysis due to missing data. Nearly one third of the original sample had missing data for the OH-SE. This was a result of the response option of “I never feel like this”, treated as a missing response. The original scale from which ours was derived did not include this option. It was added in our study based on feedback from the expert and Aboriginal advisory groups. The second reason for missing data was the OH-F scale, as an option of “I don’t know”, also treated as a missing response, also added on the advice of the expert and Aboriginal reference groups. Validation of the OH-SE scale [27] involved assessing sociodemographic differences between those with and without scale scores. There were differences by age group, but no differences were identified for other sociodemographic variables. We theorised a number of reasons for the high number of participants choosing the option of “I feel like this” including literacy levels and social stigma around depression and anxiety, with participants opting out as a more socially desirable response, particularly in the younger age group. The decision was made to proceed for this study with a complete case sample for all analyses to reduce the risk of misinterpreting the results of multivariable analyses with different number of participants depending on the confounders used in each model. Despite the smaller sample size, clear associations between OH-SE and both measures of oral health were identified, suggesting that OH-SE is an important factor to investigate further for this community. This study involved a convenience sample of Aboriginal adults in a regional location, so extrapolation of results to the broader Australian population needs to be made with caution. Although we hypothesised causal pathways between OH-SE and oral health outcomes to drive analysis, this is a cross-sectional study and causation cannot be inferred.

Despite these weaknesses, this study has key strengths and is an important addition to the sparse literature investigating psychosocial factors and oral health outcomes for Aboriginal people in Australia. The fact that 400 Aboriginal adults in a regional location completed baseline questionnaires involving questions pertaining to psychosocial factors, with a complete data set for over 250 participants, is a successful study outcome. This indicates the cultural acceptability of the survey instruments and study design. The inclusion of a broad range of sociodemographic variables known to be associated with general and oral health outcomes for Aboriginal people ensured these factors were not explaining the association between OH-SE and oral health outcomes.

## Conclusion

Lower levels of OH-SE were associated with a higher prevalence of SROH and greater impacts of oral health among Aboriginal adults in regional South Australia. These associations persisted after controlling for sociodemographic and general and oral health-specific psychosocial confounders. Perceived stress resulted in the most significant attenuation in the association between OH-SE and oral health outcomes. The findings indicate that self-efficacy beliefs may provide an opportunity for intervention to improve oral health outcomes for Aboriginal adults in regional South Australia.

## Data Availability

The datasets generated and analysed during the current study are not publicly available due the sensitive nature of questionnaire information for the study community but are available from the corresponding author on reasonable request.

## Declarations

All methods were carried out in accordance with relevant guidelines and regulations.
